# Sampling of *An.gambiae s.s *mosquitoes using Limburger cheese, heat and moisture as baits in a homemade trap

**DOI:** 10.1186/1756-0500-4-284

**Published:** 2011-08-11

**Authors:** Eunice Anyango Owino

**Affiliations:** 1 School of Biological Sciences, University of Nairobi, P.O. Box 30197-00100, Nairobi, Kenya

## Abstract

**Background:**

Ample evidence has shown that odour baited traps are likely to provide an objective monitoring tool for the host-seeking fraction of mosquito vectors of diseases like malaria and bancroftian filariasis. Such traps could eventually become part of primary healthcare systems used to study the vector biology and epidemiology of mosquito-borne diseases. I hereby, report a study that sampled *Anopheles gambiae sensu stricto *mosquitoes in a screen house using a homemade trap baited with a combination of Limburger cheese and moisture, Limburger cheese and heat, or Limburger cheese, moisture and heat.

**Findings:**

Tests on the efficacy of the developed trap to sample *An. gambiae s.s*, mosquitoes using Limburger cheese, moisture and heat as baits were carried out in a screen house measuring 11.4 × 7.1 × 2.8 m. The studies were done in three phases. In the first phase the efficacy of the trap to sample *An. gambiae s.s*. using odour and moisture was tested. The second phase was to test the efficacy of the trap to sample *An. gambiae s.s*. using Limburger cheese and heat. In the third phase a combination of Limburger cheese, moisture and heat was tested. Tests were carried out for 27 consecutive nights.

The designed trap collected a total of 59 *An. gambiae s.s*. in three trials. The trap baited with Limburger cheese and moisture collected 7 *An. gambiae s.s *in 7 days. The mean catch per day was 1. The trap baited with Limburger cheese and heat collected zero *An. gambiae s.s *in 11 days. The mean catch per day was therefore 0. The trap baited with Limburger cheese, moisture and heat collected 52 mosquitoes in 27 days and the mean catch was 1.93.

**Conclusions:**

This study indicates that a non-electric fan driven trap baited with a combination of Limburger cheese, heat and moisture has a potential as an effective sampling tool for the malaria vector, *Anopheles gambiae s.s*. However, further optimization studies would be necessary.

## Background

Ample evidence has shown that odour baited traps are likely [1-3] to provide an objective monitoring tool for the host-seeking fraction of mosquito vectors of diseases like malaria and bancroftian filariasis. Such traps could eventually become part of primary healthcare systems used to study the vector biology and epidemiology of mosquito-borne diseases, knowledge of which is vital for planning and assessing outcome of intervention strategies. One might even foresee the development of odour baited mosquito traps [1,2] that might be used to reduce the vector population in a village or in an individual's bedroom to divert mosquitoes away from occupants. Such traps could then be used in the trapping of mosquitoes in large numbers [2]. This would lead to the control of infection and transmission rates of diseases spread by mosquitoes like malaria, rift valley fever and bancroftian filariasis. Current efforts centre on searching for new attractants and attractant formulations [1-3] and developing odour baited trapping devices [2-5]. Efficacy trials under field [6] and semi-field conditions have also been carried out [7].

The discovery by Knols *et al *in 1996 that the odour of Limburger cheese-which to the human nose has a smell that is strongly reminiscent of human foot odour-was highly attractive to *An. gambiae s.s *in wind tunnel bioassays [8] together with the discovery of Van den Hurk *et al *in 1997 [9] which showed that it is possible to sample an appropriate fraction of mosquitoes with a non-suction trap baited with human odour provide important steps towards the development of a cost effective sampling, monitoring and possibly an alternative method for controlling malaria vectors and malarial illness in Africa.

Therefore, in an effort to develop an effective and a cheap odour baited trap that does not operate on the principle of an electric fan and which could be used in sampling and controlling of disease spreading mosquitoes, efficacy studies using a homemade trap were done. The trap was baited with Limburger cheese and moisture, Limburger cheese and heat or Limburger cheese, moisture and heat.

## Semi Field Investigations

The semi field investigation reported was conducted inside a screen house located at the Thomas Odhiambo campus of ICIPE in Mbita point town council, Suba District, Western Kenya.

### Trap design

A prototype mosquito odour baited trap was thus developed and evaluated as described below

**Figure 1:**
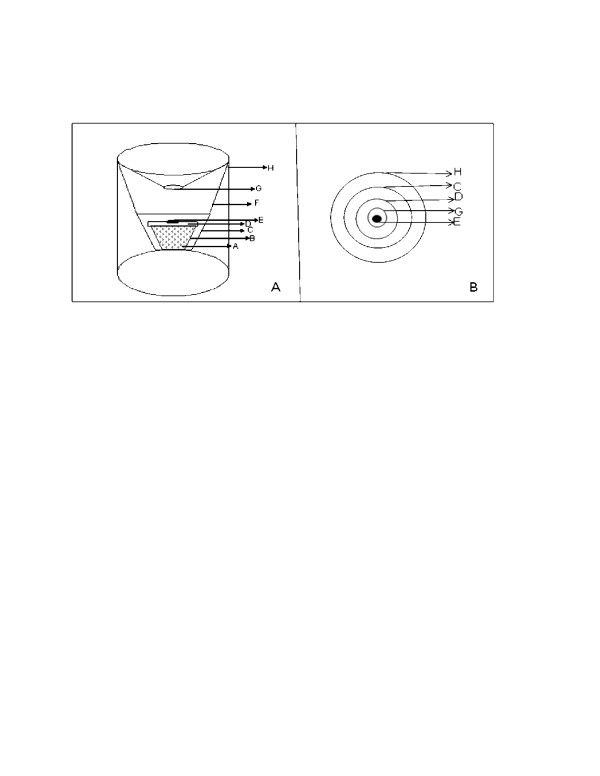
**Trapping Device**. Panel A: Side view of the trapping device with the traditional insulated container fitted at the bottom and placed inside a plastic bucket. Panel B: Top view of the trapping device with the traditional insulated container fitted at the bottom and placed inside a plastic bucket. A, water in a plastic container; B, Plastic container; C, Insulated container; D, lid of the plastic container; E, Limburger cheese on a filter paper; F, lower part (net) of the trapping device; G, aperture (entrance for mosquitoes) at the upper part of the trapping device, H, bucket.

**Figure 2:**
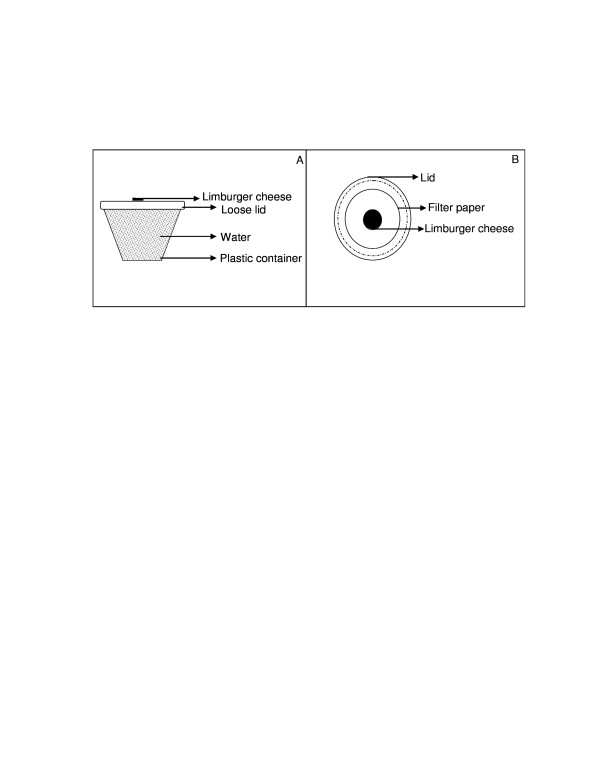
**Limburger cheese on plastic containter**. Panel A: Side view of Limburger cheese put on top of the lid of a plastic container filled with warm water. Panel B: Top view of Limburger cheese on top of the lid of the plastic container.

The prototype trap was made of four components; (I) a plastic container covered with a loose lid and filled with water, (ii) an insulated container, (iii) a trapping device, and (IV) a plastic bucket. All these are shown in Figure 1. The purpose of the water was to provide moisture and heat (when using warm water) to the trap. On top of the lid of the plastic container was put a filter paper with 0.6 g of Limburger cheese placed at its center (Figure 2). This arrangement ensured that the heat from the warm water melted the Limburger cheese which was the odour source. The arrangement also ensured that the heat provided convectional currents which acted as a medium in which heat, moisture and odour were carried upwards to the entrance of the trap from where the cues could presumably be perceived by mosquitoes released in the surrounding area.

The plastic container filled with warm water and Limburger cheese on filter paper placed on the lid were put inside a traditional insulated container from the *abagusii *community of Western Kenya. The insulated container was made from reeds plastered with cow dung and can preserve warmth for more than ten hours. The purpose of the insulated container was to provide insulation to the plastic container that had warm water so that heat was not lost from the trap quickly. The plastic container was put in the insulated container in such a way that its bottom was in contact with the container (Figure 2). The insulated container was then fitted in a trapping device whose upper part was made of a metal framework covered with mosquito netting material such that this upper part appeared conical in shape with an aperture at the center. The lower part of the trapping device was left open. It is into this opening at the bottom of the trapping device that the insulated container was fitted (Figure 2). The purpose of the aperture on the upper part of the device was to act as an entrance for mosquitoes into the trap. The diameter of the cone-like shape of the upper part of the trapping device was 35 cm and that of the aperture was 5 cm. The height of the trapping device from the bottom side to the aperture at the cone like shape was 28 cm.

All of these components of the trap were then put into, a plastic bucket (Figure 2) whose diameter and height were 35 cm and 38 cm respectively. The purpose of this bucket was to prevent further loss of heat, odour and moisture from the trap to the surrounding and instead channel these cues towards the entrance (aperture) so that the cues could be perceived by the mosquitoes and the mosquitoes could be attracted and enter the trap through the aperture.

### Tests

**Figure 3:**
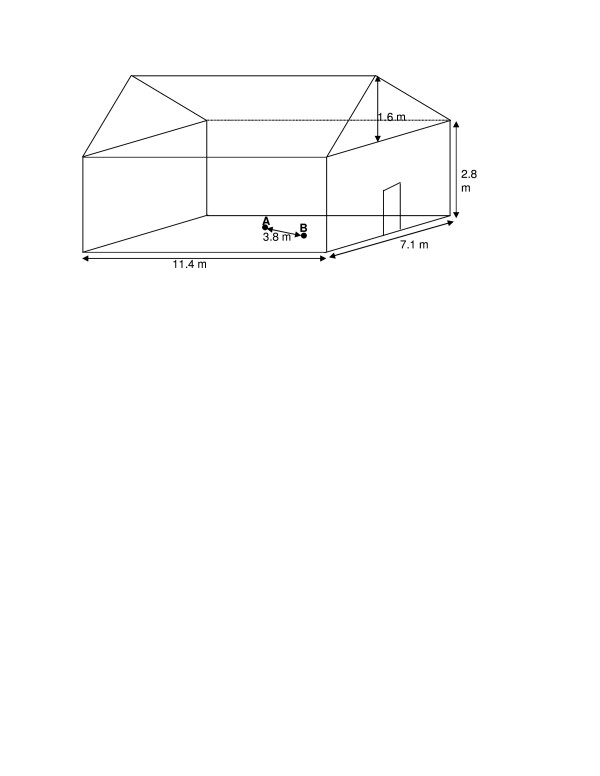
**Screen house where semi-field experiments were conducted**. Point A is the position at which the odour baited trap was placed and point B is the point at which the paper cup containing mosquitoes was put 3.8 m away from the odour baited trap.

The efficacy of the developed trap to sample *An. gambiae s.s*, mosquitoes using Limburger cheese, moisture and heat as baits was tested in a screen house measuring 11.4 × 7.1 × 2.8 m (Cambridge Glass House Co. Ltd., UK) (Figure 3). The screen house was modified by replacing all glass parts with dark-green netting (density 90%) permitting airflow and moisture to enter the system. A sliding door provided entrance into the screen house; the door had a double layer of similar netting to prevent escape of released mosquitoes and entry of wild ones.

The Limburger cheese used in these experiments was imported from the Netherlands and was stored in the refrigerator at 6°Celsius while not in use.

Strains of *An. gambiae s.s*. which were originally from Mbita Point, Western Kenya were obtained from the campuses insectary where they have been maintained since January 2001.

One hundred (100) female *An. gambiae s.s *in standard 30 × 30 × 30 cm cage were starved for six hours from 13.45 to 19.45 hrs. Only cotton wool soaked in pure water was put on top of the cage to provide moisture in the trap so that the mosquitoes could not get dehydrated. The mosquitoes were then collected into a paper cup using an oral aspirator and the mouth of the paper cup was sealed with a piece of cotton wool. The odour baited trap was placed at the center of the screen house and the paper cup was placed 3.8 m away (Figure 3). The cotton wool at the mouth of the paper cup was removed to release the mosquitoes from 22.00 to 06.00 hours. Thereafter the aperture on the trap was sealed with cotton wool. The trapping device was then removed from the bucket and the mosquitoes collected were counted and the numbers recorded.

The studies were done in three phases. In the first phase the efficacy of the trap to sample *An. gambiae s.s*. using Limburger cheese and moisture as cues was tested. The trap was set as earlier described except that the plastic container was filled with cold water instead of warm water so that only moisture from the cold water and odour from Limburger cheese could be perceived by the experimental mosquitoes released in the screen house. This study was carried out for 7 consecutive nights.

The second phase was to test the efficacy of the trap to sample *An. gambiae s.s*. using Limburger cheese and heat. The trap was set as earlier described except that the plastic container carrying warm water was covered with a tight lid so that only heat from the warm water and odour from Limburger cheese but not the moisture could be perceived by the experimental mosquitoes released in the screen house. The studies were conducted for 11 consecutive nights.

The third phase was to test the efficacy of the trap to sample *An. gambiae *using Limburger cheese, moisture and heat cues. The trap was set as earlier described so that heat, moisture and odour of Limburger cheese could be perceived by the experimental mosquitoes released in the screen house. Tests were carried out for 27 consecutive nights.

### Statistical analysis

Data were analyzed using PASW Statistic 11.5 (SPSS version 11). The mean mosquito catch per trap per night were first calculated and compared using tables. Data analysis was done using independent samples t test whereby the mean of catches in the three trials was compared. Unequal variance was assumed. The effect of stimuli was considered to be significant when P < 0.05

## Results

The designed trap collected a total of 59 *An. gambiae s.s*. in three trials. The trap baited with Limburger cheese and moisture collected 7 *An. gambiae s.s*. in 7 days. The mean catch per day was 1. The trap baited with Limburger cheese and heat collected zero *An. gambiae s.s *in 11 days. The mean catch per day was therefore 0. The trap baited with Limburger cheese, moisture and heat, collected 52 *An. gambiae s.s*. in 27 days and the mean catch was 1.93. These are presented on Table 1 below. The mean catch of *An. gambiae s.s *mosquitoes collected by the trap baited with Limburger cheese, moisture and heat was significantly higher than that of *An. gambiae s.s*. in collected by the trap baited with Limburger cheese and heat only (F = 3.834,D.F = 26(2), P = 0.001, t _0.05_) while the mean catch of *An. gambiae s.s*. collected by the trap baited with moisture and Limburger cheese only was not significantly different from that of *An. gambiae s.s*. in collected by the trap baited with Limburger cheese, moisture and heat (F = 0. 460, P = 0.465, t _0.05_, D.F = 11 (2)).

**Table 1:** The total and mean catch of mosquitoes collected by the homemade trap baited with Limburger cheese, moisture and heat in three trials at the screen house

Trial	Stimulus				Catches	
	Limburger cheese	Moisture	heat	N	n	Mean
1	Present	present	absent	7	7	1^ab^
2	Present	absent	present	11	0	0^b^
3	present	present	present	27	52	1.93^a^

N represents the number of days and n, the total number of mosquitoes trapped

Values following each other in the same column with different letter superscripts are significantly different at (P = 0.05).

## Discussions

When the trap was baited with Limburger cheese (an odour), heat and moisture (physical stimuli) it collected a higher number of *An. gambiae s.s *than when it was baited with either Limburger cheese and moisture or Limburger cheese and heat. This observation is supported by earlier observations that host-seeking mosquitoes are exposed to a wide variety of visual, olfactory, gustatory and physical stimuli and a combination of these stimuli act as cues for host identification and location [10-12].

Wright in 1975 [13] described mosquito attraction to a human arm as a response to warmth and humidity while Limburger cheese, which has a smell reminiscent to foot odour to the human nose, was observed to attract *An. gambiae s.s *in a wind tunnel bioassay [8]. One would therefore, suggest that there was a synergistic effect amongst the attractants that lead to high catches.

One would also imagine that a combination of odour, heat and moisture simulates a living human host more than a combination of odour and moisture or a combination of odour and heat hence the higher numbers of *An. gambiae s.s *which has been observed to be highly anthropophilic [14].

It would also be important to mention that Olanga *et al *2010 found that olfactory cues are the key mediators of the mosquito host-seeking process and that heat and moisture play a minor role [3].

The trap as described in this study with the various combinations of baits has shown a very low response rate. This could have been due to the fact that it was not operating on the principle of a fan that either produces an active air current sucking mosquitoes into the trap [15] or pumps out an odour laden air current out of the trap thus guiding mosquitoes towards the trap [16].

It would also be prudent to mention that future optimization of the trap and the baits and even combination of these baits with other baits that have been used to successfully sample mosquitoes [1] could lead to a trap that could effectively be used in sampling, monitoring and controlling malaria vectors.

## Competing interests

The author declares that they have no competing interests.

## Authors' contributions

EAO conceived, designed, performed the experiments and analyzed the data.
